# Apical Extrusion of Debris Following Root Canal Preparation With ProTaper Universal, Reciproc Blue, and WaveOne Rotary Systems: An In Vitro Study

**DOI:** 10.1155/ijod/8097838

**Published:** 2025-11-23

**Authors:** Mansour Jafarzadeh, Pegah Sarraf, Samira Shahsiah, Narges K. H. Almasi, Mohammad Hossein Nekoofar

**Affiliations:** ^1^Department of Endodontics, School of Dentistry, Ahvaz Jundishapur University of Medical Sciences, Ahvaz, Iran; ^2^Department of Endodontics, School of Dentistry, Tehran University of Medical Sciences, Tehran, Iran; ^3^School of Advanced Technologies in Medicine, Tehran University of Medical Sciences, Tehran, Iran; ^4^Department of Endodontic, Bahçeşehir University School of Dentistry, İstanbul, Türkiye

**Keywords:** apical debris extrusion, endodontics, instrumentation, ProTaper Universal, reciproc, root canal preparation, WaveOne

## Abstract

**Background and Objectives:**

Cleaning and shaping is an important step in root canal treatment. Apical extrusion of debris in the process of root canal instrumentation can cause inflammation and lead to treatment failure; therefore, it should be minimized. This study aimed to compare the apical extrusion of debris following root canal instrumentation with ProTaper Universal (PTU), Reciproc Blue (RB), and WaveOne (WO) rotary systems.

**Materials and Methods:**

This in vitro, experimental study was conducted on 45 anterior single-rooted, single-canal sound teeth with one apical foramen. An access cavity was prepared by a fissure diamond bur, and apical patency was ensured by a #10 K-file. Working length was determined 1 mm shorter than the apex. All teeth were then randomly distributed into three groups of PTU, RB, and WO and underwent root canal instrumentation with the respective systems. Apically extruded debris was collected in pre-weighed Eppendorf tubes during root canal instrumentation. The weight of apically extruded debris was calculated by subtracting the weight of tubes before instrumentation from their weight after instrumentation. Data were analyzed by SPSS version 26 through one-way ANOVA and Tukey's test.

**Results:**

The lowest extrusion of debris occurred in the WO group (9.087 ± 2.004 µg) followed by PTU (13.809 ± 2.424 µg) and RB (19.177 ± 7.661 µg) (*p* < 0.001). Apical extrusion of debris in the WO group was significantly lower than that in PTU (*p*=0.025) and RB (*p* < 0.001). Apical extrusion of debris in PTU was significantly lower than that in RB (*p*=0.01).

**Conclusion:**

All rotary systems caused apical extrusion of debris. However, WO resulted in less apical extrusion of debris than PTU and RB.

## 1. Introduction

Root canal preparation stands as a pivotal step in endodontic therapy, underlining the criticality of achieving thorough debridement to eradicate residual bacteria and inflammatory byproducts [1–4]. However, this process can inadvertently introduce debris into the periradicular space, potentially triggering periapical inflammation and postoperative flare-ups [5–8]. Mitigating the apical extrusion of debris assumes paramount importance in minimizing inflammatory reactions and fostering optimal healing outcomes [9]. Various factors, including the instrumentation technique, file type, and design, exert significant influence on the extent of debris extrusion [5, 10, 11].

Notably, rotary nickel–titanium (NiTi) files have emerged as preferred alternatives to manual techniques, with the crown-down approach demonstrating superior efficacy in minimizing debris extrusion [11, 12]. In contrast, reciprocating motion, characterized by alternating clockwise and counterclockwise rotations, offers a distinct technique for root canal preparation [10].

Among the widely utilized NiTi files, the ProTaper Universal (PTU) and WaveOne (WO) systems have garnered considerable attention [11]. While these systems enhance procedural efficiency, the persistent challenge of instrument fracture persists, prompting ongoing efforts to enhance their physical and mechanical properties [13]. Recent innovations, such as the Reciproc Blue (RB) and WO systems, constructed from the resilient M-wire NiTi alloy, offer heightened flexibility and resistance to fracture, potentially mitigating procedural complications [14]. Furthermore, the thermal treatment process employed in the manufacture of RB files facilitates the formation of a blue-colored titanium oxide layer, enhancing their durability and performance [14]. There are contradictory findings regarding apical extrusion according to different systems and motions. Some studies reported greater extrusion with reciprocating motion compared to continuous rotation [15, 16], while others found the opposite or were unable to determine which motion leads to more debris extrusion [17, 18].

 The present study aims to compare and evaluate the apical extrusion of debris following root canal preparation using the PTU multifile triangular design, the RB S-shaped single-file, and the WO systems S-shaped single-file, with a specific focus on rotary and reciprocation motion. This comprehensive assessment aims to furnish dental clinicians with valuable insights into the comparative efficacy of these systems, aiding in the informed selection of an optimal approach tailored to individual patient needs [5].

## 2. Materials and Methods

This in vitro, experimental study was conducted at the Endodontics Department of the School of Dentistry of Ahwaz University of Medical Sciences. A total of 45 extracted sound single-rooted, single-canal anterior teeth with mature apices and canal curvature of 0°–10° were evaluated in this study. The study was approved by Ahwaz JundiShapur University of Medical Sciences (Ethical Approval Code: IR.AJUMS.REC.1400.125).

According to the effect size reported by Nevares et al. [19] a sample size calculation with a 5% significance level and 90% power indicated a minimum of 15 specimens per group.

The teeth were selected by convenience sampling. All teeth had one single apical foramen, completely formed apices, no root resorption or caries, and 0°–10° root canal curvature. The number of roots and apical foramina was ensured by taking buccolingual and mesiodistal radiographs. The canal curvature was determined on buccolingual radiographs according to the Schneider's method [20]. The measurements were made by AutoCAD software.

Soft tissue residues were removed from the root surface by a periodontal curette. The teeth were then immersed in 5.25% sodium hypochlorite for 1 h. To standardize the teeth, the crowns were cut by a high-speed handpiece and a diamond bur such that 15 mm of root length remained. An access cavity was prepared by a diamond fissure bur (Kaveh, Iran), and apical patency of all canals was ensured by a #10 K-file (Dentsply, Maillefer, North America). The working length was determined 1 mm shorter than the apex. All teeth were then randomly distributed into three groups and stored in 1% sodium hypochlorite (Samen Darou, Iran) until use. Prior to the experiment, the teeth were immersed in distilled water (DaruPakhsh, Iran) for 20 h.

The root canals were cleaned and shaped by RB, PTU, and WO rotary files as instructed by the manufacturers. Apical flaring was continued in all three groups up to the #25 instrument size. The same operator, following manufacturers' instructions, performed instrumentation of all root canals. Each rotary instrument was discarded after flaring of three canals. In the PTU group, S1, S2, F1, and F2 files were used to the working length [21]. In the RB group, the #25/8% file was used. In the WO group, the #25/8% file was used with gentle in-and-out movement. Collection of apically extruded debris was performed according to the method described in a previous study [22]. For this purpose, Eppendorf tubes were weighed by a digital scale (Sartorius Analytical, Germany) with 0.0001 g accuracy. Three consecutive measurements were made, and the mean weight of each tube was calculated and recorded. The teeth were placed in the tubes to the level of their cementoenamel junction and fixed with cyanoacrylate glue to prevent the leakage of irrigating solution. A needle was inserted through the stopper to equalize the internal and external pressure.

For root canal irrigation after using each instrument, a total volume of 4 mL of distilled water was injected into the canal by a double side-vented 30-gauge needle (Helma Teb, Tehran, Iran) with maximum penetration depth of the needle without being locked in the canal with an in-and-out motion. Each tooth was then removed from the tube, and the root surface was rinsed with 1 mL of distilled water inside the tube to collect debris adhering to the root surface in the tube. The tubes were subsequently placed in an incubator at 70°C for 5 days to allow the distilled water to evaporate before measuring the weight of the dry debris. A second examiner, unaware of the group assignments, conducted the weight calculations. Using the same analytical balance, the Eppendorf tubes were weighed to determine the total weight, including the extruded debris. Three successive weighings were performed for each tube. The dry weight of the extruded debris was determined by subtracting the weight of the empty tube from that of the tube containing debris.

Data were analyzed by SPSS version 26. The Kolmogorov–Smirnov test was applied to analyze the normality of data distribution. The amount of apically extruded debris was compared among the three groups by one-way ANOVA followed by the Tukey's test.

## 3. Results

The Kolmogorov–Smirnov test confirmed normal distribution of data in all three groups (*p* > 0.05). Minimum debris extrusion was recorded in WO (9.087 ± 2.004 µg) followed by the PTU (13.809 ± 2.424 µg) and RB (19.177 ± 7.661 µg) group. According to one-way ANOVA, the three groups were significantly different regarding apical extrusion of debris (*p* < 0.001, Table 1).

According to Tukey's test, apical extrusion of debris in WO group was significantly lower than that in PTU (*p*=0.025) and RB (*p* < 0.001). Apical extrusion of debris in PTU was significantly lower than that in RB (*p*=0.01).

## 4. Discussion

All tested endodontic instrumentation systems have shown some degrees of apical extrusion of debris [1, 22, 23]. Some systems have lower and some others have higher extrusion of debris. Such variations in the amount of extruded debris are important in the level of post-endodontic pain because the systems with greater apical extrusion of debris increase the risk of flare-ups [24]. On the other hand, production and apical extrusion of debris is one of the most important causes of post-endodontic inflammation and symptomatic apical periodontitis [11]. This study aimed to compare the apical extrusion of debris by PTU, RB, and WO. A systematic review and meta-analysis by Caviedes-Bucheli et al. [10] showed that the inflammatory reactions caused by apical extrusion of debris were influenced by not only the number of files but also their type of movement and instrument design. The present results revealed the lowest apical extrusion of debris by WO (reciprocal movement), followed by PTU (rotary movement) and RB (reciprocal movement). A systematic review by Zhang et al. [11] reported minimum extrusion of debris by ProTaper Next and WO, with no significant difference between them; their results were somehow in line with the present findings.

In the present study, apical extrusion of debris by the WO single-file system was significantly lower than that by PTU (multifile system) and RB. The reciprocating movements push the debris apically, while rotary movements push the debris coronally [25, 26]. Nonetheless, in the present study, the two instruments with reciprocating movements yielded significantly different amounts of apically extruded debris. Aside from the instrument movement, many other factors can affect apical extrusion of debris, such as instrument core mass, cross-sectional design of the instrument, and final apical diameter [25, 27, 28]. Each instrument used in the present study has a different cross-sectional design. RB files have an S-shaped cross-section. PTU files have a convex triangular cross-section, and WO files have a variable cross-sectional design such that the file has a radial land cross-sectional design close to the tip, while its cross-sectional design changes from a convex triangle modified with radial land to a rake angle with a convex triangular shape at the middle of the working blade and close to the shaft [29]. Ozsu et al. [22] reported that the amount of apically extruded debris in the process of instrumentation with the WO single-file system was lower than the PTU multifile system in single-rooted single-canal mandibular premolars. Keskin and Sarıyılmaz [25] found that the amount of apically extruded debris and irrigants was lower in WO Gold and ProTaper Next than RB in mandibular premolars. Also, another study reported higher apical extrusion of debris by PT than WO, although this difference was not statistically significant [24]. The reason may be that in use of WO instruments, reciprocation aims to mimic the balanced force kinematic technique, and applies less pressure to push the debris apically [24]. Another study, however, reported contradictory results and showed that ProTaper Next generated lower amounts of debris than WO [11].

The present study demonstrated that PTU resulted in significantly lower apical extrusion of debris compared to RB. Among the three systems, RB caused the highest debris extrusion, which contrasts with Nouroloyouni's findings [30]. This discrepancy may be attributed to the different types of teeth used in the two studies. In the present study, anterior teeth with larger apical foramina were selected, potentially leading to more debris extrusion. Aside from the use of thermomechanical alloys, RB instruments are fabricated similar to their previous generation and operate with the same reciprocating parameters. Thermomechanical procedures improve the mechanical properties of the instruments, such as their flexibility and resistance against cyclic fatigue [25]. It has been reported that Reciproc [26] and RB [24, 25] result in higher apical extrusion of debris compared with ProTaper Next. On the contrary, Koçak et al. [31] reported that apical extrusion of debris by Reciproc was significantly lower than that by ProTaper F2 in mandibular premolars. Controversy in the results can be due to differences in root length and diameter and volumetric size of the teeth.

The available literature indicates apical extrusion of debris by all systems, irrespective of the adopted technique. Also, even in the use of the same technique, the weight of the extruded debris varies across different studies. A number of factors can affect apical extrusion of debris, such as type of teeth, complexity of the root canal system, working length, instrumentation technique, kinematics of the instruments used [32, 33], apical enlargement, formation of dentin plug, type, and amount of irrigating solution used and method of delivery of irrigant into the root canal [10, 11]. Working length is an influential factor on production of apical debris. Working length 1 mm shorter than the apical foramen can significantly decrease the amount of apically extruded debris [11]. Thus, the working length was determined 1 mm shorter than the apical foramen in the present study. The type of irrigant can also affect the amount of extruded debris and success of root canal therapy [11]. Although sodium hypochlorite is the most commonly used irrigant, a neutral irrigant was used in the present study because the focus was on the physics of movement of instruments [34]. Distilled water was used as an irrigant in the present study since it does not increase the weight of apically extruded debris [11].

The technique of collection and weighing of the extruded debris is another important topic. The collected debris should be created only by root canal instrumentation. They should also be dehydrated to obtain their actual weight. Different methods have been proposed for this purpose. The technique proposed by Myers and Montgomery [35] has higher advantages compared with other quantitative techniques because it allows separate quantification of extruded debris and irrigant. However, this technique has drawbacks as well, such as the absence of an apical barrier. Moreover, sensitivity of the analytical scale is important for quantification of apically extruded debris in this technique. Since the environmental conditions are not controlled in this technique, the apically extruded debris can become hydrated due to exposure to humid air. Also, the technique of drying of apically extruded debris is affected by external factors, such as temperature and moisture [11]. Another limitation is that this study was an in-vito study and clinical variables such as periapical tissue resistance, host immune response, and irrigant dynamics under physiological conditions could not reproduced. In addition, the use of extracted anterior teeth with relatively large apical foramina may have influenced the amount of extruded debris and limits direct extrapolation to posterior teeth with narrower and more complex canals. Future studies using different tooth types and incorporating in vivo models could provide results that are more clinically relevant.

## 5. Conclusion

The current results showed the lowest apical extrusion of debris by WO, followed by PTU and RB. WO caused significantly lower extrusion of debris than the other two systems. Future studies are required to compare other techniques and assess the effects of anatomical variations, different irrigants, and the presence of a sound apical barrier versus the presence of periapical lesions on the results.

## Figures and Tables

**Table 1:** Apical extrusion of debris (µg) by WO, PTU, and RB systems.

Rotary system	Mean and standard deviation	Minimum	Maximum	*p*-Value*⁣*^*∗*^
WaveOne (*n* = 15)	9.087 ± 2.0049	6.18	12.52	<0.001
ProTaper Universal (*n* = 15)	13.908 ± 2.424	10.40	16.44	
Reciproc Blue (*n* = 15)	19.177 ± 7.661	8.38	37.47	

^*∗*^One-way ANOVA.

## Data Availability

The data used to support the findings of this study are included within the article.
